# Magnetic Suppression of Perceptual Accuracy Is Not Reduced in Visual Snow Syndrome

**DOI:** 10.3389/fneur.2021.658857

**Published:** 2021-05-04

**Authors:** Ozan E. Eren, Ruth Ruscheweyh, Veronika Rauschel, Thomas Eggert, Christoph J. Schankin, Andreas Straube

**Affiliations:** ^1^Department of Neurology, Ludwig Maximilians University of Munich, University Hospital - Großhadern, Munich, Germany; ^2^Department of Neurology, Inselspital, Bern University Hospital, University of Bern, Bern, Switzerland

**Keywords:** visual snow syndrome, cortical hyperexcitability, magnetic suppression of perceptual accuracy, migraine, pathophysiology

## Abstract

**Objective:** Patients with visual snow syndrome (VSS) suffer from continuous (“TV snow-like”) visual disturbance of unknown pathoetiology. In VSS, changes in cortical excitability in the primary visual cortex and the visual association cortex are discussed, with recent imaging studies tending to point to higher-order visual areas. Migraine, especially migraine with aura, is a common comorbidity. In chronic migraine and episodic migraine with aura but not in episodic migraine without aura, a reduced magnetic suppression of perceptual accuracy (MSPA) reflects a probably reduced inhibition of the primary visual cortex. Here we investigated the inhibition of the primary visual cortex using MSPA in patients with VSS, comparing that with MSPA in controls matched for episodic migraine.

**Methods:** Seventeen patients with VSS were compared to 17 age- and migraine-matched controls. Visual accuracy was assessed by letter recognition and modulated by transcranial magnetic stimulation delivered to the occipital cortex at different intervals with respect to the letter presentation (40, 100, and 190 ms).

**Results:** Suppression of visual accuracy at the 100-ms interval was present without significant differences between VSS patients and age- and migraine-matched controls (percentage of correctly recognized trigrams, control: 46.4 ± 34.3; VSS: 52.5 ± 25.4, *p* = 0.56).

**Conclusions:** In contrast to migraine with aura, occipital cortex inhibition, as assessed with MSPA, may not be affected in VSS.

## Introduction

Patients with visual snow (VS) describe continuous, mostly black and white tiny flickering dots in their entire visual field, comparable to the old TV-static noise when missing the analog signal. When accompanied by other visual symptoms such as afterimages (palinopsia), impaired night vision (nyctalopia), or increased light sensitivity (photophobia), it is called visual snow syndrome (VSS) (1–3). Its pathophysiology is still under discussion, and although it is highly associated with migraine with and without aura and may partially overlap with these, recent research strongly suggests that VS is a distinct disorder (2–5). The visual disturbance sums up to a clinical picture that is best explained by dysfunction of the higher-order visual cortex. Consistently, fluorodeoxyglucose-positron emission tomography (PET) investigations showed hypermetabolism in the lingual gyrus, an area of the higher visual association cortex (3, 6). Importantly, these findings from functional neuroimaging were confirmed by voxel-based morphometry by two independent groups which demonstrated increased gray matter volume in the same cortical area (6, 7). A possible neurophysiological correlate of the involvement of higher visual areas could be the significantly prolonged latency of the late N145 potentials with normal P100 potentials in visual evoked potentials (4). However, the picture is likely more complex with studies pointing to a dysfunction of the primary visual cortex, considering thalamocortical dysrhythmia as the origin of VSS (8, 9) and demonstrating alterations also in non-visual, acoustic, and limbic areas (6).

Here we used magnetic suppression of perceptual accuracy (MSPA) to further elucidate the role of the primary visual cortex or at least its inhibition in VSS. Reduced MSPA reflects reduced inhibition of the primary visual cortex, which is seen in chronic migraine and episodic migraine with aura, but not in episodic migraine without aura (10). Reduced MSPA in VSS would therefore argue for a decreased local inhibition of the primary visual cortex similar to episodic migraine with aura.

## Materials and Methods

The study was conducted in accordance with the Declaration of Helsinki and approved by the local ethics committee (227-15). All patients gave written informed consent. The results of the study were presented in preliminary format at the International Headache Conference 2017 (11).

### Subjects

For recruitment, the study was advertised in social media with support from the self-help group on VS, “Eye on Vision Foundation” (http://www.eyeonvision.org/). We first assessed the eligibility of interested patients during telephone interviews conducted by a headache specialist familiar with VSS. The interview was cross-checked by a second headache specialist. Inclusion criteria were age ≥ 18 years and presence of VSS (subtype black and white dots) in accordance with the criteria published previously (2). Exclusion criterion was intake of any illicit drugs currently or within 2 weeks prior to the onset of VSS. Brain MRI was normal in all subjects. Later, the VSS patients were examined at presentation by one of the two mentioned specialists. Of medications known to possibly affect cortical excitability, only one patient was on lamotrigine and three patients were on mirtazapine. Travel expenses were reimbursed, and no further payment was made for study participation. Patients with VSS were compared to age- and migraine-matched subjects.

### Measurement of MSPA

MSPA was measured according to our previous work (10). To summarize, three-letter sequences (so-called trigrams) were presented for 30 ms on a monitor in front of the subjects. They were instructed to read the letters aloud from left to right. In a first step, training runs were performed to adjust contrast in a manner that ~80% of the letters could be recognized correctly by the subject without transcranial magnetic stimulation (TMS) intervention. In a second step, the experiment was started by presenting a series of 54 trigrams, followed each by a TMS pulse (output of at least 70% of the possible maximum output, Magstim 200, The MagStim Company Ltd, Whitland, UK) *via* a 90-mm circular coil to the occipital cortex in randomized intervals of 40, 100, or 190 ms in regard to the trigram presentation. The time between the start of trigram presentation and TMS pulse delivery is called stimulus onset asynchrony (SOA). Later, the percentage of correctly recognized trigrams was calculated for each SOA interval. All subjects were measured interictally; as corroborated by telephone contact, no subject reported a migraine attack or aura within 2 days after the experiment.

### Statistical Analysis

Statistical analysis was done using SPSS 25 (IBM Corp. Released 2017. IBM SPSS Statistics for Windows, version 25.0.0.1, 32-bit-version, Armonk, NY: IBM Corp.). Statistical significance was assumed at *p* ≤ 0.05. The demographics of the groups were compared using chi-square test.

ANOVA was used for MSPA comparison (within-subject factor: SOA, between-subject factor: group). Where ANOVA was significant (*p* ≤ 0.05), *t*-test with Bonferroni correction was used for *post hoc* analysis.

### Data Availability Statement

Anonymized data will be shared at request from any qualified investigator.

## Results

### Subjects

Seventeen patients with visual snow syndrome (six females and 11 males; mean age, 30.0 ± 10.8 years; 12 with comorbid migraine, seven of them also with typical migraine aura) were compared to 17 control subjects (C) (14 females and three males; mean age, 28.3 ± 8.2 years; 12 with comorbid migraine, none of them with typical migraine aura). The groups did differ in gender (χ = 7.77 *p* = 0.005) and aura (χ = 8.82 *p* = 0.003), but not in migraine (χ = 0, *p* = 1) and age χ = 16.87, *p* = 0.66. For more information on study population, see the Supplementary Table 1. If not stated otherwise, the term “controls” describes the group of age- and migraine-matched subjects.

### MSPA

**Figure 1:**
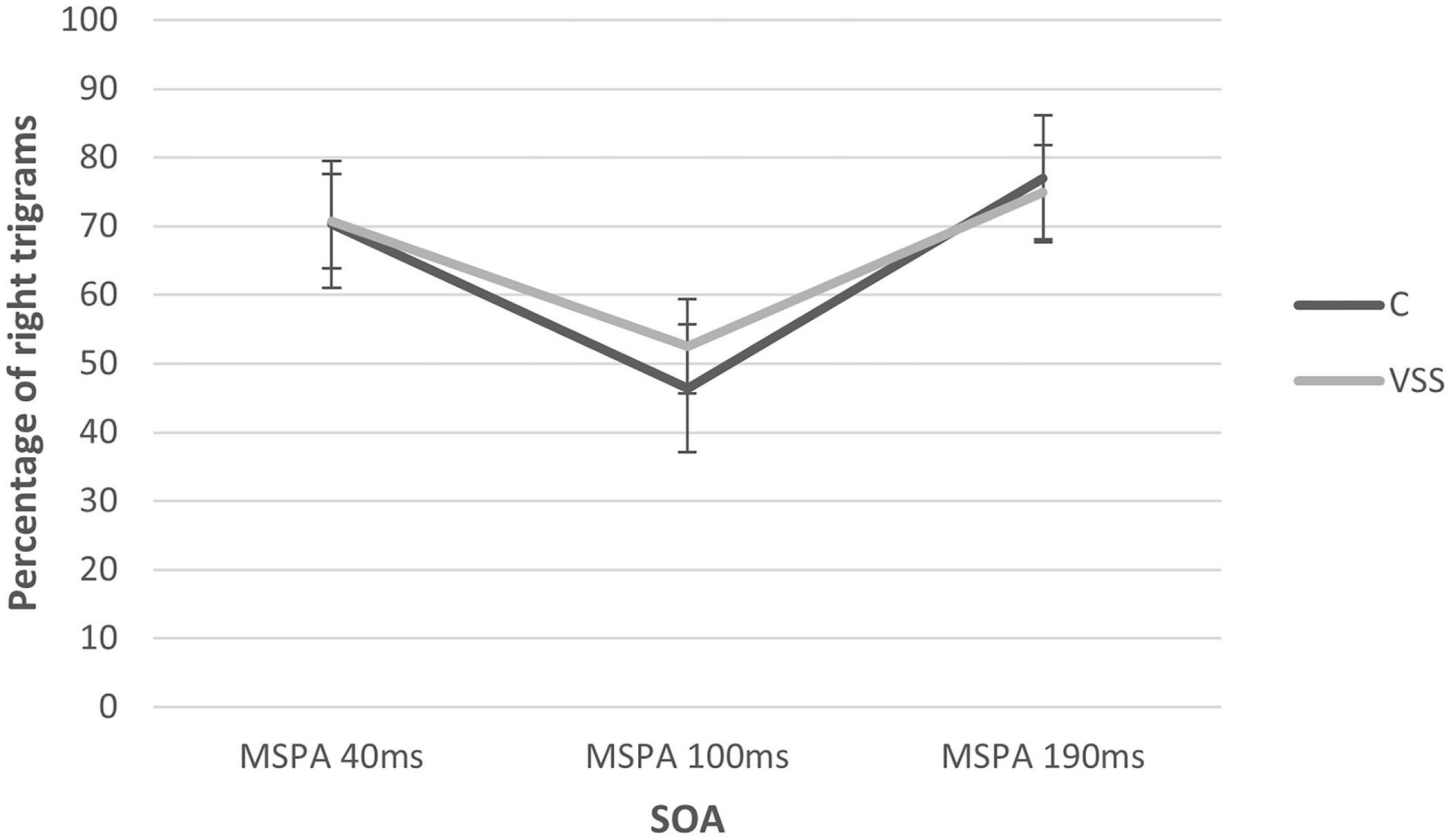
MSPA profiles of subjects with visual snow syndrome and migraine-matched control group at the three SOA intervals. There was a significant change with the SOA interval but no group difference. SOA, stimulus onset asynchrony.

The MSPA profiles of patients with VSS and migraine-matched controls can be seen in Figure 1 and Table 1.

**Table 1:** Mean percentage of correctly detected trigrams at the three different stimulus onset asynchrony (SOA) intervals (40, 100 and 190 ms).

**SOA**	**Average percentage correct (mean** **±** **SD)**	
	**Visual snow syndrome**	**Control**
40 ms	70.70 ± 14.88	70.26 ± 26.53
100 ms	52.51 ± 25.41	46.40 ± 34.26
190 ms	74.95 ± 10.08	76.91 ± 22.62

There was a significant main effect of SOA interval (rmANOVA: Greenhouse–Geisser *F* 31.75, *p* ≤ 0.01) with a significant suppression of visual accuracy at 100 ms compared to 40 and 190 ms. There was no significant main effect of group (rmANOVA: *F* 0.70, *p* = 0.47). Additionally, we performed an explorative subgroup analysis beginning with comparing the same control group only with VSS patients without migraine aura, showing almost identical percentages of correctly recognized trigrams at 100 ms (C: 46.40 ± 34.26 vs. VSS: 46.67 ± 27.99), again without significant group differences (rmANOVA: *F* 0.261, *p* = 0.77).

Afterwards, to understand the effect of aura in our VSS group itself, we compared VSS patients with migraine with aura (VSSMwA; *n* = 7) and VSS patients with migraine without aura (VSSMwoA; *n* = 5). There was again a significant main effect of SOA interval (rmANOVA: Greenhouse–Geisser *F* 6.31, *p* ≤ 0.05) with a significant suppression of visual accuracy at 100 ms (VSSMwoA: 48.2 ± 31.89 vs. VSSMwA: 60.85 ± 20.21) compared to 40 and 190 ms, but also no significant main effect of group (rmANOVA: *F* 0.40, *p* = 0.57).

## Discussion

The main result of this study is that magnetic suppression of perceptual accuracy is not reduced in visual snow syndrome when compared to controls matched for migraine. In this respect, VSS differs from migraine with aura (12). The significant suppression at 100 ms is comparable instead to that of patients with migraine without aura and controls without migraine (10). Previous work of Aurora and Mulleners showed that healthy controls exhibited the largest suppression at 100 ms SOA, followed by migraine patients without aura, while chronic migraine patients and episodic migraineurs with aura showed the least suppression (12, 13). Consistently, within the VSS group, subjects with migraine with aura showed a smaller MSPA compared to those without aura. However, the difference was not significant, maybe due to the small sample sizes in the subgroups.

It has been discussed that a reduced MSPA reflects a higher cortical excitability due to a deficiency of intracortical inhibition of the primary visual cortex (12). This would facilitate the initiation of cortical spreading depression, resulting in an attack of migraine with visual aura, but apparently playing a minor role in migraineurs without aura (10, 14).

Visual snow syndrome is thought to involve cortical hyperexcitability or a lack of inhibition. The present results suggest that, at least for our collective, such hyperexcitability does not seem to arise from the primary visual cortex. From the clinical description, the typical visual phenomena seem to be best explained by a dysfunction of higher-order visual cortex. This is supported by overlapping morphological and functional correlates in the visual association cortex in PET and MRI (3, 6) as well as alterations in the late visual evoked potentials (4).

## Limitations

One limitation of the study is the lack of matching for gender, but to the best of our knowledge, there is no evidence of sex differences in MSPA. Nevertheless, an influence cannot be excluded. Another limitation is the lack of matching for aura, but based on our explorative subgroup analysis irrespective of inclusion or exclusion of the aura patients in the VSS group, the results remained unchanged. Additionally, it should be mentioned that, in the VSS group, the disability caused by headache measured by Migraine Disability Assessment was lower compared to the migraine-matched controls, as we matched for comorbidity and not severity. Lastly, we could have added a healthy control group without comorbid migraine and give more details on clinical data.

## Conclusion

This study demonstrates that magnetic suppression of perceptual accuracy, in contrast to the situation in migraine with aura, is not reduced in VSS compared to migraine-matched controls. Therefore, although hyperexcitability apparently occurs in both VSS and migraine aura, the locations seem to be different. The primary visual cortex might not be the main location in VSS.

## Data Availability Statement

The raw data supporting the conclusions of this article will be made available by the authors, without undue reservation.

## Ethics Statement

The studies involving human participants were reviewed and approved by Ludwig Maximilians University Munich (227-15). The patients/participants provided their written informed consent to participate in this study.

## Author Contributions

AS, CS, OE, RR, TE, and VR contributed to the conception and design of the study. CS, OE, RR, and VR contributed to the acquisition and analysis of data. AS, CS, and OE contributed to the drafting of a significant portion of the manuscript. All authors contributed to the article and approved the submitted version.

## Conflict of Interest

OE reports grants from Friedrich-Baur Foundation, Deutsche Migräne- und Kopfschmerzgesellschaft during the conduct of the study and Novartis outside the submitted work. RR reports personal fees and/or other from Allergan, Novartis, Teva Pharmaceuticals, Hormosan outside the submitted work. CS reports grants from Deutsche Migräne- und Kopfschmerzgesellschaft, Eye on Vision Foundation, and Baasch Medicus Foundation during the conduct of the study, personal fees from Novartis, Eli Lilly, Allergan, Almirall, Amgen, MindMed, and Grünenthal, and personal fees and other from Teva Pharmaceuticals outside the submitted work. AS reports personal fees from Allergan, Bayer, Sanofi, Desitin, Electrocore, Eli Lilly, and Teva Pharmaceuticals and grants from the German Research Council, Kröner-Fresenius Foundation, Ludwig-Maximilian University, and Friedrich-Baur Foundation outside the submitted work. The remaining authors declare that the research was conducted in the absence of any commercial or financial relationships that could be construed as a potential conflict of interest.
