# Supplementary material for: Magnetic Suppression of Perceptual Accuracy Is Not Reduced in Visual Snow Syndrome
Source: Front Neurol. 2021 May 4;12:658857. doi: 10.3389/fneur.2021.658857 (PMC8129492; doi:10.3389/fneur.2021.658857)
Supplement: Supplementary file 1 [file Table_1.DOCX]

|  | VSS patients | Migraine matched controls | Statistics | df | p |
| --- | --- | --- | --- | --- | --- |
|  | n = 17 | n = 17 |  |  |  |
| Age | 30.0±10.8 | 28.3±8.2 | *T*=-0.52 | 32 | 0.61 |
| Sex female | 14 | 6 | χ^2^=7.77 | 1 | 0.005 |
| Migraine | 12 | 12 | χ^2^=0 | 1 | 1 |
| Aura | 7 | 0 | χ^2^=8.82 | 1 | 0.003 |
| MIDAS score | 1.2±2.4 | 11.2±12.3 | U=46 |  | 0.001 |
| Palinopsia  (trailing, afterimages) | 64.7% | | | | |
| Entoptic phenomena  (floaters, BFEP, photopsia) | 94.1% | | | | |
| Nyctalopia | 70.6% | | | | |
| Photophobia | 88.2% | | | | |

MIDAS: Migraine Disability Assessment Score, BFEP: blue field entoptic phenomenon
